# Supplementary material for: Characterization of DAG Binding to TRPC Channels by Target-Dependent cis–trans Isomerization of OptoDArG
Source: Biomolecules. 2022 Jun 7;12(6):799. doi: 10.3390/biom12060799 (PMC9221121; doi:10.3390/biom12060799)
Supplement: Supplementary file 1 [file biomolecules-12-00799-s001.zip › biomolecules-1737758-supplementary.pdf]

biomolecules

MDPI

Supplemantary Material for

Characterization of DAG binding to TRPC channels by target-dependent cis-trans isomerization of OptoDArG

Hazel Erkan-Candag [1], Denis Krivic [1,2], Mathias Gsell [1], Mina Aleksanyan [2,3], Thomas Stockner [4] Rumiana Dimova [2], Oleksandra Tiapko [1,*] and Klaus Groschner [1,*]

[1] Gottfried Schatz Research Center - Biophysics, Medical University of Graz, Graz, Austria
[2] Max Planck Institute of Colloids and Interfaces, Potsdam, Germany
[3] Institute for Chemistry and Biochemistry, Freie Universität Berlin, Berlin, Germany
[4] Institute of Pharmacology, Medical University of Vienna, Vienna, Austria
*Correspondence: oleksandra.tiapko@medunigraz.at; klaus.groschner@medunigraz.at; Tel.: +43 316 316 385 71500

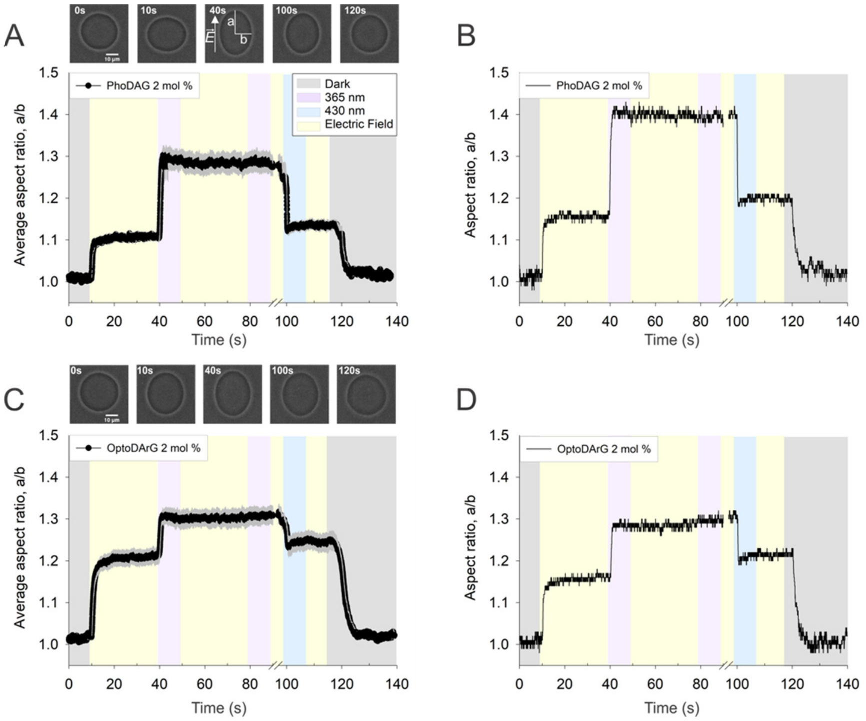

Figure S1. Assessing light-induced membrane expansion via electrodeformation of GUVs containing (A, B) 2 mol % PhoDAG-1 ot (C, D) 2 mol % OptoDArG. (A, C) Data averaged over 9 vesicles and (B, D) examples for single-GUV experiments. The initial and final experimental configurations, shaded in grey represent dark state with no electric field (quasi-spherical vesicle, storing excess area in fluctuations), the effect of electric field (in dark) is shaded in yellow (fluctuations are pulled out and the vesicle deformation can be precisely measured), while the purple and blue shaded regions illustrate the duration (10 s) of UV and blue light irradiation (while the electric field is on), respectively. Electric field is applied from 10 s to 120 s and the onset of UV light is at 40 s and 80 s and of blue light at 100 s. The images above panels (A) and (C) show phase contrast screenshots of electrodeformed GUVs . The semiaxis a and b defining the aspect ratio a/b are indicated on the second image. The gray-shaded scatter in (A, C) show the standard error of mean (n = 9). The scale bar is 10 µm.

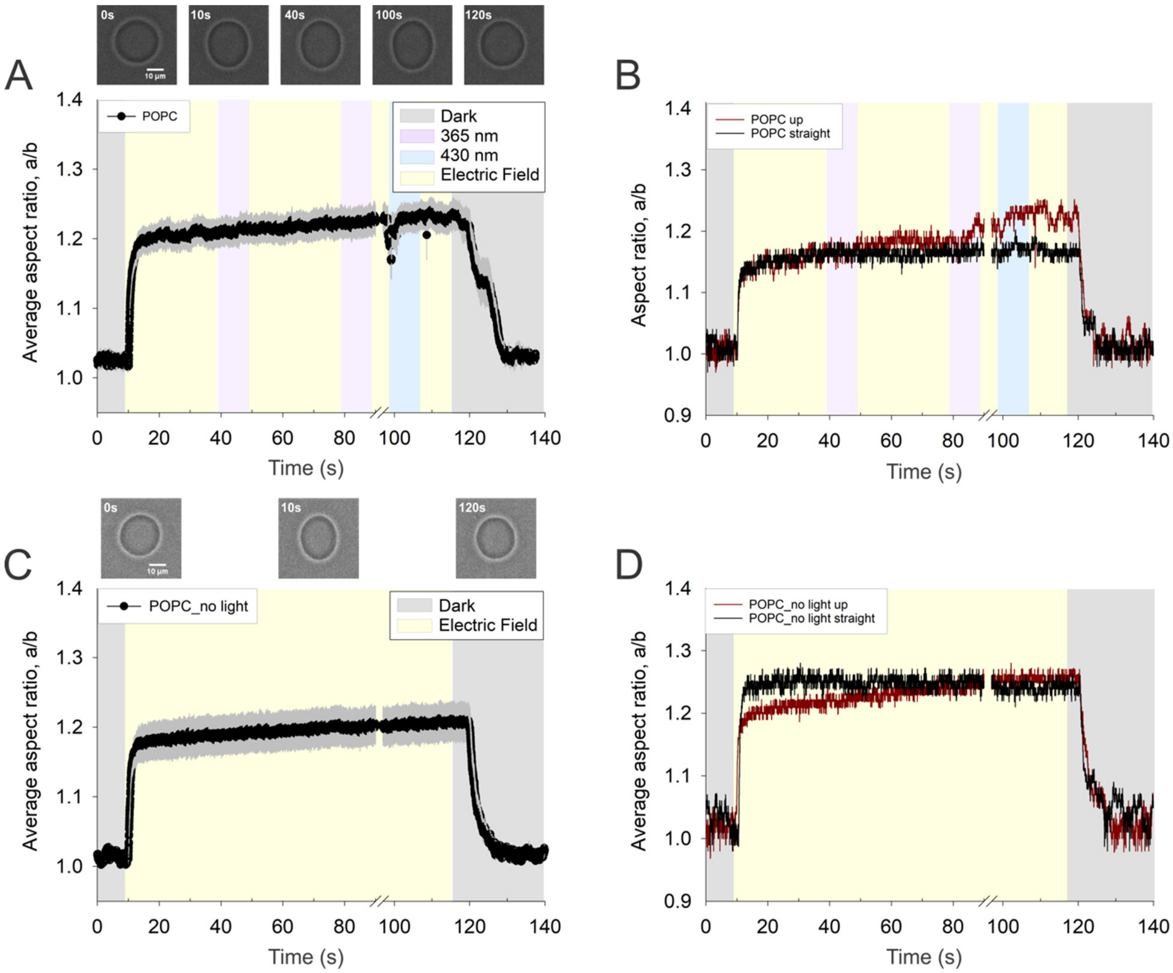

Figure S2. Control experiment showing electrodeformation of pure POPC GUVs in the presence (A, B) and absence (C, D) of UV/blue light. Light protocol is identical to the one used in Supplementary Figure 3. Protocol not featuring UV/blue light illumination had only electric field applied from 10 s to 120 s. Data in (A) and (C) are averaged respectively over 13 and 12 vesicles and the gray scatter shows standard error of mean and (B, D) show two examples of individual vesicles (red and black traces) with steady and growing trend, the latter resulting from pulled out submicroscopic deffects. In the absence of light (C, D), there was no filter change, but the x-axis change was retained to facilitate the comparison of results. The snapshots above (A) and (C) show the GUV shape change at each turnpoint of the protocol. The scale bar is 10 μm.

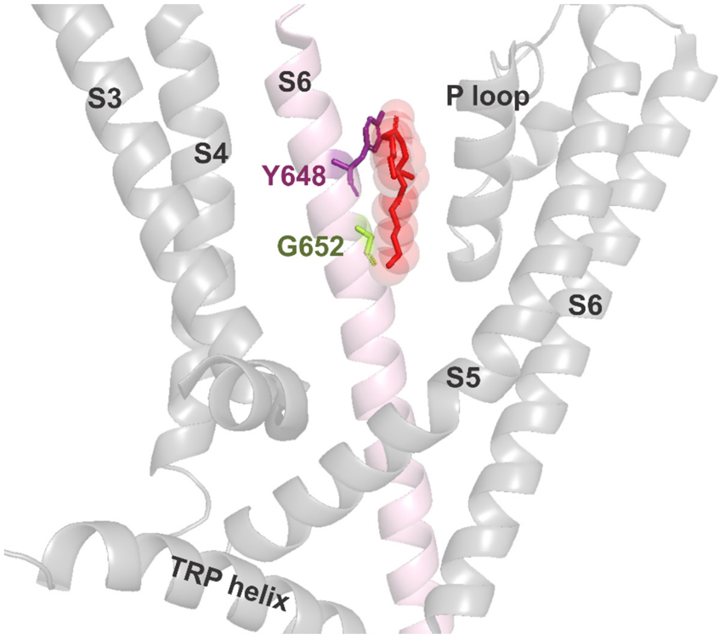

Figure S3. L2 binding site structure of TRPC3 (hTRPC3 PDB ID: 6CUD; (Fan et al, 2018)). TRPC3 subunit a is shown in grey, with S6 of subunit b is highlighted in pink (formed L2 binding site). Red represents DAG density.

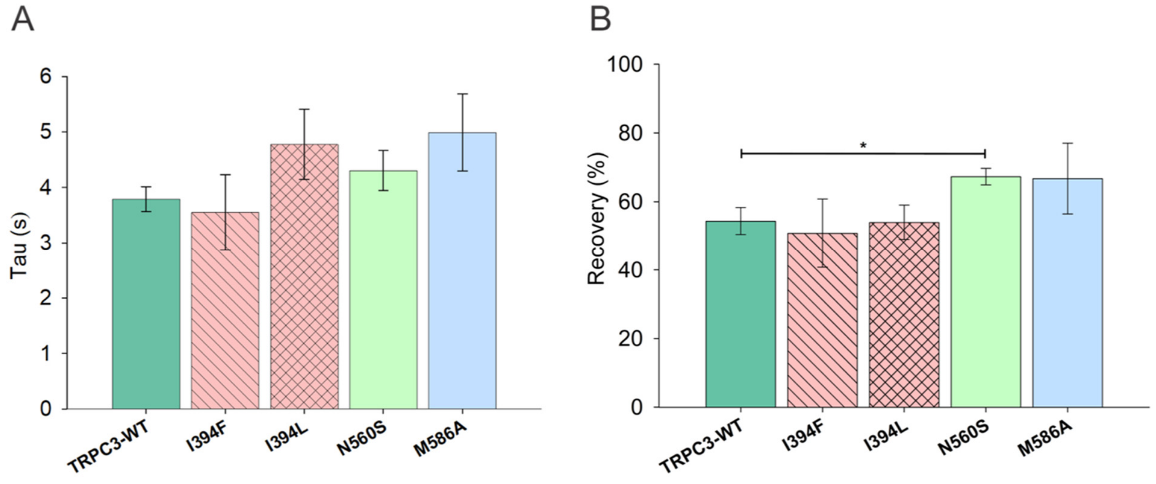

Figure S4. Mutations in L1 region do not alter thermal relaxation of OptoDArG-mediated deactivation kinetics in the dark. Bar charts illustrating deactivation time constant (Tau; A) and percentage of the recovery to basal conductance after UV was switched off (Dark; B) TRPC3-WT and TRPC3 mutants. Number of biological repetitions for each condition ≥ 6. Data are mean ± S.E.M.; two-tailed t-test or Mann-Whitney test were applied; *$P < 0.05$.
